# Sleep Pattern Is Related to Mental Health among Chinese Collegiate Student Athletes

**DOI:** 10.3390/ijerph19158961

**Published:** 2022-07-23

**Authors:** Wei Wei, Weimin Liu

**Affiliations:** 1 School of Physical Education, Central China Normal University, 152 Luoyu Road, Wuhan 430079, China; 2 Department of Physical Education, Hohai University, 1 Xikang Road, Nanjing 210098, China

**Keywords:** sleep duration, napping, college student, athlete, anxiety, depression

## Abstract

Regarding the association between sleep and napping duration and mental health in young and older adults, some studies indicated a positive association, while others indicated a negative, or no, association between them. Moreover, collegiate student athletes have different mental health stressors, such as training pressure, improving sports performance, and relationships with coaches. Therefore, sleep is important for athletes. Whether sleep duration is related to their mental health is unclear. Thus, this study aimed to examine the association between nighttime sleep duration, daytime napping duration, and mental health among collegiate student athletees. This cross-sectional study included 700 college athletes. Sleep and daytime napping durations were assessed using a self-reported questionnaire. The Zung Self-rating Depression Scale and Generalized Anxiety Disorder-7 assessed mental health. A multivariate logistic regression analysis was conducted to examine the adjusted association between sleep duration and mental health. In this study, the odds ratios for depression and anxiety symptoms were significantly higher for short sleep duration (<7 h). Additionally, a significant positive association was found between daytime napping duration and the prevalence of depression. This study indicates that short nighttime sleep and long daytime napping duration may be risk factors for collegiate student athletes’ mental health, having important implications for educators and coaches.

## 1. Introduction

The burden of mental disorders continues to increase, with significant health and economic consequences worldwide [1]. In China, the prevalence of mental disorder was 9.3% in a 2015 survey [2]. This was a dramatic increase from 1982 (1.3%) [3] to 1993 (1.4%) [4] in China. Mental disorders are related to negative health outcomes, such as cardiovascular disease [5], diabetes [6], disability [7], and suicide [8]. Moreover, mental illnesses are more common among university students than other population groups [9]. Previous studies have revealed that the frequency of anxiety disorders in athletes is comparable to the general population [10]. Additionally, the higher prevalence of mental health symptoms and disorders in athletes versus the general population may be related to sport-specific stressors [11]. Symptoms range in severity and may include feeling down, decreased motivation, fatigue, difficulty concentrating, or suicidal ideation [12]. These outcomes not only influence student athletes’ health, but also their academic or sports performance. Therefore, improving mental health and preventing mental disorders in collegiate student athletes is crucial. Factors such as physical activity and nutrition intake are considered preventive and alleviating factors for mental disorders. However, athletes already have a higher physical activity and relative high nutrition intake than other population. Thus, it may not be appropriate to improve their mental health by these means. Meanwhile, sleep is an indispensable part of people’s daily life. Athletes also need to obtain enough physical recovery through sleep. Additionally, improved sleep quality and restful sleep are associated with the absence of depressive symptoms [13,14]. Therefore, sleep may be an optimal way to improve athletes’ mental health.

Sleep is a homeostatic process that involves an active and periodic biological state crucial for good physical and mental health [15]. Sleep durations that are too short or too long have been associated with many negative health outcomes. Having 7–8 h of nighttime sleep is recommended as the best range for health, which is related to a reduced risk of coronary heart disease [16] and hypertension [17]. Moreover, the risk of death was lowest in those with an average of 7–8 h of nighttime sleep [18]. Some studies have suggested that daytime napping is associated with an increased risk of diseases such as hypertension [19] and Type 2 diabetes [20]. These diseases are risk factors for mental disorders. It is well known that sufficient sleep can physically relax people and improve mood. Insufficient sleep can provoke an inflammation increasing response via increased cytokine secretion [21], and studies show that inflammatory markers are associated with depression [22,23]. In addition, neurotransmitter serotonin plays a crucial role for effect of sleep and mental health [24,25]. Therefore, it could be considered that nighttime sleep duration and daytime napping duration may affect mental health.

On the basis of extant literature, some studies, such as an American study and two Chinese studies, investigated this association and showed that short sleep duration at night is associated with a higher risk of mental disorders [26,27,28]. However, other studies indicated that there is a U-curve association between sleep duration and mental health, showing that 6–9 h’ sleep duration is associated with lowered risk of mental disorders [29,30]. In addition, the findings of previous studies examining the association between daytime napping duration and mental health are also not consistent. Some studies indicate that shorter napping duration is better for mental health [31,32], while others indicate that longer napping duration is better [28,33]. Sleep patterns and mental health status in collegiate student athletes differ from those of other populations. Additionally, most students in Chinese universities live in dormitories, which is a collective lifestyle specific to Chinese university students. Therefore, it is worth examining the association of sleep and daytime napping durations with mental health specifically in Chinese collegiate student athletes.

For this purpose, we designed a cross-sectional study to investigate the association between nighttime sleep duration, daytime nap duration, and mental health in Chinese collegiate student athletes. Considering that short sleep duration deprives student athletes of sufficient rest, prolonged nighttime sleep or napping duration may lead student athletes to skip meals, or lower their training or study time, which may correlate with higher risk of depression. Therefore, we hypothesized that 7–8 h of nighttime sleep and a short daytime nap duration would be associated with better mental health.

## 2. Materials and Methods

### 2.1. Participants

The collegiate student athletes in this study were recruited from the school of physical education of five universities in the northern cities of China in 2019. We invited 800 student athletes to participate in this study. Participants completed the questionnaire in their classrooms before class under the guidance of the instructors. Of the invited participants, 761 agreed to participate and provided informed consent for their data to be analyzed. This study was approved by the Human Research Ethics Committee of Haerbin Sport University. Athletes were excluded if they did not attend the practice session when the questionnaire was administered (*n* = 22), or if some of the responses to the questions were missing (*n* = 39). Following these exclusions, the final study population comprised 700 athletes (380 males and 320 females).

### 2.2. Assessment of Sleep Status

Sleep status was assessed using a questionnaire that included questions related to sleep. Information on sleep duration was cited from Pittsburgh Sleep Quality Index (PQSI): (1) “During the past month, what time did you usually go to bed at night?”; (2) “During the past month, how long (in minutes) did it usually take you to fall asleep each night?”; (3) “During the past month, at what time did you usually get up in the morning?” [34]. Sleep duration was divided into three categories: <7 h, 7–8 h, and >8 h, and the final sleep duration was calculated using three questions. First, information on daytime nap duration was obtained by asking “During the past month, did you have a daytime nap habit?”. The possible answers were “yes” or “no”. If participants chose “yes”, then they were asked “How long is your typical nap?”. Participants replied with “___hours, ___minutes”. We divided daytime nap duration into four categories: (1) shortest (≤30 min/day), (2) (31–60 min/day), (3) (61–90 min/day), and (4) longest (>90 min/day).

### 2.3. Assessment of Mental Health

#### 2.3.1. Depression

The presence of depression was assessed in the participants, and the degree of depression was also assessed using the Chinese version of the Zung Self-rating Depression Scale (SDS) [35] which is widely used in Chinese studies [36,37,38]. The SDS is a self-report, 20-question instrument designed to screen for depression in large patient groups and to measure the severity of depression. Each question is rated on a four-point scale from 1 (rarely) to 4 (most of the time). The SDS generates a total score ranging between 20 and 80, with good internal consistency and validity [39]. Based on previous studies [40], a cutoff score of ≥40 was used to classify students with depression in this study. The test score reliability coefficient for SDS was 0.70 using Cronbach’s alpha.

#### 2.3.2. Anxiety

We used the Generalized Anxiety Disorder-7 (GAD-7) to evaluate the severity of anxiety symptoms. It is a validated scale [41], which consists of seven items with total scores ranging from 0 to 21. Higher scores indicate more severe symptoms. GAD-7 was widely used in Chinese studies [42,43,44], and previous studies have shown that severity in the GAD-7 is represented by scores ≥5, ≥10, and ≥15 for mild, moderate, and severe levels of anxiety symptoms, respectively [45,46]. We used a cutoff score of ≥5 to define students with mild anxiety symptoms. The test score reliability coefficient for GAD-7 was 0.94 using Cronbach’s alpha.

### 2.4. Covariates

Basic information was obtained using a questionnaire that included sex (male or female), grade (freshman, sophomore, senior, or junior), and race (Han nationality or minority race). BMI was calculated as weight (kg)/height (m)^2^. Living expenses were categorized as low, medium, and high (CNY < 1000, 1000–1499, and >1499 per month, respectively). Based on their smoking status, the participants were assessed as smokers or nonsmokers. According to their drinking habits, they were classified as daily drinker, occasional drinkers, and nondrinkers. Considering the small number of participants in the drinking daily category (*n* = 3), we combined daily drinkers and occasional drinkers into a single “drinker” category. Thus, drinking habit was defined as drinking or nondrinking. We assessed academic pressure and pressure from the coach as none, a little, or high. The “a little” and “high” categories were combined into one “yes” category, owing to the small number of participants in the high category (*n* = 6). Sustained pain was evaluated by the question “Do you have sustained pain caused by a sports injury, not including delayed onset muscle soreness?”. We evaluated subjective training fatigue as not tired, relatively tired, or very tired. Finally, years of exercise means how many years student athletes have been engaged in sports, and was divided into “<3 years” and “≥3 years”.

### 2.5. Statistical Analyses

A multivariate logistic regression analysis was used to examine the association between sleep status and mental health. Data are presented as odds ratios with 95% confidence intervals (95% CIs). Sleep status and mental health were the independent and dependent variables, respectively. The differences in mental health variables (depression and anxiety) in participants’ characteristics were examined by t-test for continuous variables or by the x^2^ test for categorical variables. We analyzed the association between sleep status and mental health in the unadjusted and adjusted models. Model 1 was adjusted for basic characteristics (sex, grade, race, and BMI), and Model 2 was adjusted for lifestyle- and training-related factors (the items in Model 1, plus living expenses, smoking, drinking habits, academic pressure, sustained pain, pressure from the coach, and subjective fatigue). All trend p-values were calculated using the categories of sleep status (sleep duration and nap duration), and intergroup p-values were also shown in the main results. A statistical difference was defined as significant when the p-value was <0.05. All statistical analyses were performed using the IBM Statistical Package for the Social Sciences (SPSS) Version 22.0 (IBM, Armonk, NY, USA).

## 3. Results

The basic data used in this study were obtained from 700 participants. Of these, 178 (25.4%) were classified as having depression and 291 (41.6%) as having symptoms of anxiety. The characteristics of the depression and anxiety groups are shown in Table 1. The proportion of participants who were males, had medium living expenses, had no academic pressure, were relatively tired from training, had 7–8 h of daily sleep duration, or had short napping duration was higher in the no-depression category. Conversely, the proportion of participants who were of a minority race, had little or high academic pressure, had pressure from coaches, no tiredness from training, <7 h of daily sleep duration, or relatively long napping duration was higher in the depression category. Meanwhile, the proportion of junior students who had no academic pressure or had 7–8 h of daily sleep duration was higher in the no-anxiety category. Conversely, the proportion of participants who were freshmen and sophomores or had a little academic pressure or had <7 h of daily sleep duration was higher in the anxiety category. Notably, daytime nap duration was longer among those with anxiety symptoms.

Table 2 presents the adjusted associations between sleep duration and depression and anxiety symptoms. No linear association was observed. However, those who slept less than seven hours at night had a significantly higher odds ratio than those who slept for 7–8 h for both depression and anxiety symptoms. In Model 2 (final adjusted model) of the association between sleep duration and depression, compared with the 7–8 h of sleep category, the odds ratios (95% CIs) were 1.68 (1.14, 2.48) for <7 h, and 1.66 (0.86, 3.21) for >8 h. A similar association was also found between sleep duration and anxiety symptoms. In Model 2, compared with the 7–8 h of sleep category, the odds ratios (95% CIs) were 1.46 (1.03, 2.05) for <7 h and 1.10 (0.59, 2.06) for >8 h.

Table 3 presents the adjusted associations between daytime napping duration and mental health. A positive linear association was found between daytime nap duration and prevalence of depression in the unadjusted and adjusted models. In Model 2 (final adjusted model), compared with the shortest daytime napping duration category, the odds ratios (95% CIs) of the second, third, and fourth categories were 1.20 (0.73, 1.96), 1.76 (1.03, 2.99), and 1.62 (0.99, 2.66), respectively, and trend *p* = 0.026. However, no significant association was found between daytime nap duration and anxiety symptoms.

## 4. Discussion

This study investigated the association between nighttime sleep duration, daytime napping, and mental health among collegiate student athletes. Although adjustments were made for several confounding factors, this study found that a short nighttime sleep duration (<7 h) was associated with a higher prevalence of depression and anxiety symptoms. Meanwhile, a longer duration of daytime napping was associated with a higher prevalence of depression. To our knowledge, this is the first study to examine the association between sleep duration, napping duration, and mental health among collegiate student athletes.

Previous studies have shown that the prevalence of depression among Chinese university students was 37.0% [47], 29.7% [48], and 19.4% [49]. The rates of anxiety symptoms were 24.9% [47], 53.1% [50], and 12.7% [51]. Differences in the assessment methods and cutoff points of the scales for depression and anxiety in these studies may explain these inconsistent results. Although these previous findings may not be comparable with ours, the prevalence of depression (25.4%) and anxiety symptoms (41.6%) in our study was at an average level. In contrast, two studies reported the prevalence of depression in American collegiate athletes as 21.4% [52] and 21.7% [53], which is similar to our findings.

Our findings regarding the adverse effects of short nighttime sleep duration on the incidence of depression and anxiety supported those of previous studies, for example, studies such as two American cohort studies on 15,204 and 555 adults [54,55], an American study on 4175 youths [26], and three Chinese studies on 3724 adolescents [27], 9515 university students [49], and 11,052 middle-aged and older adults [28]. All of them indicated that short nighttime sleep duration was associated with an increased risk of depressive symptoms. In addition, our finding regarding daytime napping and mental health also supported previous studies, such as a French study and a Chinese study [31,32], which indicated that there is a significantly positive association between daytime napping and depression. In summary, the difference between our study and those previous studies lies in the sample; therefore, it could be considered that it is important and meaningful that our study extends these associations to the collegiate athlete populations. Furthermore, most previous studies only examined the association between sleep duration and depressive symptoms, but did not consider napping duration and anxiety. Thus, our study strengthens the literature of the association between sleep and mental health.

However, there are also some studies that reported a U-curve association between sleep duration and mental health, suggesting that both short and long nighttime sleep duration are related to poor mental health. For example, Bao et al. indicated that, compared with those who had 8 h of nighttime sleep, people who slept <8 h and ≥9 h per night had a higher risk of depressive symptoms in 7311 Chinese adolescents [29]. Another study on 28,202 Chinese rural adults aged 18–79 years demonstrated that both shorter (<6 h) and longer sleep duration (≥10 h) were associated with an increased risk of depressive symptoms [30]. In addition, a review study also showed that both short and long sleep duration is significantly associated with increased risk of depression in adults [56]. Therefore, a U-curve association between sleep duration and depressive symptoms was also observed in our study (Table 2). However, the number of participants who slept for >8 h in our study was too small, which may explain the nonsignificant association between the long sleep duration group and the reference group. Therefore, our findings may also support those of previous studies. Regarding the association between napping duration and mental health, some studies indicated that ≥90 min of daytime napping in adult women and ≥60 min of daytime napping in middle-aged and older adults were associated with a reduced risk of depression [28,33]. A daytime napping duration of ≤30 min was also related to a lower risk of prevalent depressive symptoms in Chinese adults [57]. These previous studies were inconsistent in terms of findings regarding the association between daytime napping and depression, and further contradict our findings. This may be because of age differences. Specifically, the nighttime sleep duration of older adults is short; thus, they need a longer daytime napping duration to catch up on sleep [57]. Conversely, younger people obtain sufficient nighttime sleep compared to older people. Therefore, if student athletes engage in long daytime napping, their time for studying or training will be reduced, which may cause them to feel stressed.

We hypothesized that both short and long sleep durations are associated with poorer mental health. However, the results showed that short sleep duration, but not long sleep duration, was significantly associated with a higher risk of depression and anxiety symptoms in student athletes. This could be explained by the fact that student athletes who sleep for a short duration may have insufficient rest and a greater perceived severity of stress [58]. Perceived stress is a risk factor for mental disorders [59]. Furthermore, a study showed that university students reported a more depressed mood in the presence of a persistent pattern of short nocturnal sleep [60]. However, daytime napping can reduce nocturnal sleep duration [61]. As mentioned above, a short nighttime sleep duration is associated with depression; thus, daytime napping is related to poor mental health. In addition, high daytime sleepiness may cause loss of interest and motivation, which consequently leads to negative mental health status. From another perspective, daytime napping is associated with chronic low-grade inflammation [62], suggesting that daytime napping may be an early indicator of low-grade inflammation, predisposing individuals to depression.

This study has several limitations. First, some previous studies indicated that 6–7 h of nighttime sleep duration are beneficial for health. However, we could not include the 6–7 h sleep category because the number of participants who slept for less than six hours in this study was too low (*n* = 31). Second, causality cannot be concluded because of the cross-sectional nature of the study. Although we hypothesized that sleep duration may affect mental health, we cannot exclude the possibility that mental health status may also affect sleep duration. Third, we did not use standardized scales or objective methods to assess sleep and napping duration, such as the Pittsburgh Sleep Quality Index or sleep monitoring device, because of the limited survey time. Finally, several confounding factors were adjusted for the association between sleep duration and mental health. However, we cannot exclude the possibility that the mental health of student athletes is influenced by other factors that correlate with sleep status.

## 5. Conclusions

This study revealed that short nighttime sleep and long daytime napping durations are associated with poor mental health in collegiate student athletes. These findings provide important information in the fields of health education and preventive medicine. Paying attention to sleep status is essential for college athletes and their coaches. Prospective or randomized trial studies are needed to clarify the causality.

## Figures and Tables

**Table 1:** Basic characteristics of participants according to mental health.

	Depression		Anxiety	
	No	Yes	No	Yes
*N*	522	178	409	291
Sex (men; %)	57.1	46.1 *	50.4	59.8 *
BMI (kg/m^2^)	20.1 (19.9, 20.3)	20.0 (19.7, 20.3)	20.0 (19.8, 20.2)	20.2 (19.9, 20.4)
Grade (%)				
Freshman	28.5	26.4	21.8	36.8 ***
Sophomore	35.4	27.5	30.3	37.8 *
Junior	28.4	35.4	38.4	18.6 ***
Senior	7.7	10.7	9.5	6.9
Minority race (%)	6.5	13.5 **	7.8	8.9
Living expenses (%)				
Low	36.4	44.4	36.4	41.2
Medium	54	42.7 **	52.3	49.5
High	9.6	12.9	11.2	9.2
Nonsmoker (%)	96.9	96.1	97.3	95.9
Nondrinker (%)	75.3	75.8	76.8	73.5
Academic pressure (%)				
No	24.1	12.4 **	28.1	11.3 ***
A little	72.6	80.3 *	68.9	82.5 ***
High	3.3	7.3 *	2.9	6.2
Pressure from coaches (yes; %)	10.5	19.1 **	12.0	13.7
Sustained pain (yes; %)	9.2	12.4	9.8	10.3
Training fatigue (%)				
Not tired	10.0	18 **	11.5	12.7
Relatively tired	89.5	80.9 **	88.0	86.3
Very tired	0.6	1.1	0.5	1.0
Years of exercise (≥3 years; %)	93.3	91.6	93.9	91.4
Sleep duration (%)				
<7 h/day	44.2	54.5 *	42.5	52.9 **
7–8 h/day	47.7	34.8 **	47.9	39.5 *
>8 h/day	8.0	10.7	9.5	7.6
Daytime nap duration (%)				
1 (short)	32.6	23.6 *	32.5	27.1
2	28.2	26.4	26.9	28.9
3	15.7	23 *	18.1	16.8
4 (long)	23.6	27	22.5	27.1

Results were obtained using a *t*-test for continuous variables and *x*^2^ test for proportional variables. Values are expressed as mean (95% CIs) for continuous variables, or n (%) for categorical variables. * *p* < 0.05; ** *p* < 0.01; *** *p* < 0.001.

**Table 2:** Adjusted association between sleep duration and mental health.

	Sleep Duration			
	<7 h	7–8 h	>8 h	Trend *p*
*n*	328	311	61	
Depression (*n*)	97	62	19	
Crude	1.68 (1.17, 2.43) *	1	1.82 (0.99, 3.34)	0.171
Model 1	1.74 (1.20, 2.53) **	1	1.60 (0.85, 3.04)	0.073
Model 2	1.68 (1.14, 2.48) **	1	1.66 (0.86, 3.210	0.146
Anxiety symptoms (*n*)	154	115	22	
Crude	1.51 (1.10, 2.07) *	1	0.96 (0.54, 1.70)	0.012
Model 1	1.54 (1.11, 2.14) *	1	1.00 (1.0.55, 1.82)	0.015
Model 2	1.46 (1.03, 2.05) *	1	1.10 (0.59, 2.06)	0.070

Results were obtained using a multivariate logistic regression analysis. Values are expressed as odds ratios (95% confidential intervals). Model 1 was adjusted for sex, body mass index, grade, and race. Model 2 was further adjusted for living expenses, smoking and drinking habits, academic pressure, pressure from coaches, sustained pain, subjective fatigue, and years of exercise. * Significantly different from the 7–8 hours’ sleep duration, *p* < 0.05. ** *p* < 0.01.

**Table 3:** Adjusted association between daytime napping duration and mental health.

	Daytime Napping Duration				
	1 (Short)	2	3	4 (Long)	Trend *p*
*n*.	212	194	123	171	
Depression (*n*)	42	47	41	48	
Crude	1	1.29 (0.81, 2.07)	2.02 (1.22, 3.35)	1.58 (0.98, 2.54)	0.021
Model 1	1	1.30 (0.80,2.09)	1.87 (1.12, 3.13) *	1.62 (1.00, 2.63)	0.022
Model 2	1	1.20 (0.73, 1.96)	1.76 (1.03, 2.99) *	1.62 (0.99, 2.66)	0.026
Anxiety symptoms (*n*)	79	84	49	79	
Crude	1	1.29 (0.86, 1.91)	1.12 (0.71, 1.76)	1.45 (0.96, 2.18)	0.129
Model 1	1	1.32 (0.87, 1.99)	1.20 (0.75, 1.93)	1.47 (0.96, 2.25)	0.108
Model 2	1	1.27 (0.83, 1.94)	1.18 (0.72, 1.92)	1.51 (0.97, 2.34)	0.094

Results were obtained using multivariate logistic regression analysis. Values are expressed as odds ratios (95% confidential intervals). Model 1 was adjusted for sex, body mass index, grade, and race. Model 2 was further adjusted for living expenses, smoking and drinking habits, academic pressure, pressure from coaches, sustained pain, subjective fatigue, and years of exercise. * Significantly different from the first napping duration category, *p* < 0.05.

## Data Availability

The datasets used and analyzed during the current study are available from the corresponding author on reasonable request.

## References

[1] WHO (2019). Mental Disorders. https://www.who.int/news-room/fact-sheets/detail/mental-disorders.

[2] Huang Y., Wang Y., Wang H., Liu Z., Yu X., Yan J., Yu Y., Kou C., Xu X., Lu J. (2019). Prevalence of mental disorders in China: A cross-sectional epidemiological study. Lancet Psychiatry.

[3] Shen Y.C. (1986). Epidemiological study of mental disorders in 12 regions of China: Methodology and data analysis. Zhonghua Shen Jing Jing Shen Ke Za Zhi.

[4] Zhang W., Shen Y., Li S., Chen C., Huang Y. (1998). Epidemiological investigation on mental disorders in seven areas of China. Chin. J. Psychiatry.

[5] Theodosis-Nobelos P., Asimakopoulou E., Madianos M. (2021). Pathophysiological mechanisms of major mental disorders related to cardiovascular disease. Psychiatriki.

[6] de Jonge P., Alonso J., Stein D.J., Kiejna A., Aguilar-Gaxiola S., Viana M.C., Liu Z., O’Neill S., Bruffaerts R., Caldas-de-Almeida J.M. (2014). Associations between DSM-IV mental disorders and diabetes mellitus: A role for impulse control disorders and depression. Diabetologia.

[7] Weeks M., Garber B.G., Zamorski M.A. (2016). Disability and Mental Disorders in the Canadian Armed Forces. Can. J. Psychiatry.

[8] Chesney E., Goodwin G.M., Fazel S. (2014). Risks of all-cause and suicide mortality in mental disorders: A meta-review. World Psychiatry.

[9] WHO The WHO World Mental Health International College Student (WMH-ICS) Initiative. https://www.hcp.med.harvard.edu/wmh/college_student_survey.php.

[10] Schaal K., Tafflet M., Nassif H., Thibault V., Pichard C., Alcotte M., Guillet T., El Helou N., Berthelot G., Simon S. (2011). Psychological balance in high level athletes: Gender-based differences and sport-specific patterns. PLoS ONE.

[11] Arnold R., Fletcher D. (2012). A research synthesis and taxonomic classification of the organizational stressors encountered by sport performers. J. Sport Exerc. Psychol..

[12] American Psychiatric Association (2013). Diagnostic and Statistical Manual of Mental Disorders.

[13] Ding J., Gehrman P.R., Liu S., Yang F., Ma R., Jia Y., Yang X. (2020). Recovery Experience as the Mediating Factor in the Relationship Between Sleep Disturbance and Depressive Symptoms Among Female Nurses in Chinese Public Hospitals: A Structural Equation Modeling Analysis. Psychol. Res. Behav. Manag..

[14] Rosso A.C., Wilson O.W.A., Papalia Z., Duffey M., Kline C.E., Bopp M. (2020). Frequent restful sleep is associated with the absence of depressive symptoms and higher grade point average among college students. Sleep Health.

[15] Velayos J.L., Moleres F.J., Irujo A.M., Yllanes D., Paternain B. (2007). Anatomical basis of sleep. An. Sist. Sanit. Navar..

[16] Ayas N.T., White D.P., Manson J.E., Stampfer M.J., Speizer F.E., Malhotra A., Hu F.B. (2003). A prospective study of sleep duration and coronary heart disease in women. Arch. Intern. Med..

[17] Gottlieb D.J., Redline S., Nieto F.J., Baldwin C.M., Newman A.B., Resnick H.E., Punjabi N.M. (2006). Association of usual sleep duration with hypertension: The Sleep Heart Health Study. Sleep.

[18] Hublin C., Partinen M., Koskenvuo M., Kaprio J. (2007). Sleep and mortality: A population-based 22-year follow-up study. Sleep.

[19] Cheungpasitporn W., Thongprayoon C., Srivali N., Vijayvargiya P., Andersen C.A., Kittanamongkolchai W., Sathick I.J., Caples S.M., Erickson S.B. (2016). The effects of napping on the risk of hypertension: A systematic review and meta-analysis. J. Evid. Based Med..

[20] Chen G.C., Liu M.M., Chen L.H., Xu J.Y., Hidayat K., Li F.R., Qin L.Q. (2018). Daytime napping and risk of type 2 diabetes: A meta-analysis of prospective studies. Sleep Breath.

[21] Vgontzas A.N., Papanicolaou D.A., Bixler E.O., Lotsikas A., Zachman K., Kales A., Prolo P., Wong M.L., Licinio J., Gold P.W. (1999). Circadian interleukin-6 secretion and quantity and depth of sleep. J. Clin. Endocrinol. Metab..

[22] Dowlati Y., Herrmann N., Swardfager W., Liu H., Sham L., Reim E.K., Lanctot K.L. (2010). A meta-analysis of cytokines in major depression. Biol. Psychiatry.

[23] Howren M.B., Lamkin D.M., Suls J. (2009). Associations of depression with C-reactive protein, IL-1, and IL-6: A meta-analysis. Psychosom. Med..

[24] Ursin R. (2002). Serotonin and sleep. Sleep Med. Rev..

[25] Delgado P.L. (2000). Depression: The case for a monoamine deficiency. J. Clin. Psychiatry.

[26] Roberts R.E., Duong H.T. (2017). Is there an association between short sleep duration and adolescent anxiety disorders?. Sleep Med..

[27] Zhou T., Cheng G., Wu X., Li R., Li C., Tian G., He S., Yan Y. (2021). The Associations between Sleep Duration, Academic Pressure, and Depressive Symptoms among Chinese Adolescents: Results from China Family Panel Studies. Int. J. Environ. Res. Public Health.

[28] Li Y., Wu Y., Zhai L., Wang T., Sun Y., Zhang D. (2017). Longitudinal Association of Sleep Duration with Depressive Symptoms among Middle-aged and Older Chinese. Sci. Rep..

[29] Liu B.P., Wang X.T., Liu Z.Z., Wang Z.Y., An D., Wei Y.X., Jia C.X., Liu X. (2020). Depressive symptoms are associated with short and long sleep duration: A longitudinal study of Chinese adolescents. J. Affect. Disord..

[30] Jiang J., Li Y., Mao Z., Wang F., Huo W., Liu R., Zhang H., Tian Z., Liu X., Zhang X. (2020). Abnormal night sleep duration and poor sleep quality are independently and combinedly associated with elevated depressive symptoms in Chinese rural adults: Henan Rural Cohort. Sleep Med..

[31] Leger D., Torres M.J., Bayon V., Hercberg S., Galan P., Chennaoui M., Andreeva V.A. (2019). The association between physical and mental chronic conditions and napping. Sci. Rep..

[32] Liu Y., Peng T., Zhang S., Tang K. (2018). The relationship between depression, daytime napping, daytime dysfunction, and snoring in 0.5 million Chinese populations: Exploring the effects of socio-economic status and age. BMC Public Health.

[33] Xie B., Wang J., Li X., Zhang J., Chen M. (2020). Association between daytime napping duration and depression in middle-aged and elderly Chinese: Evidence from the China Health and Retirement Longitudinal Study (CHARLS): A cross-sectional study in China. Medicine.

[34] Buysse D.J., Reynolds C.F., Monk T.H., Berman S.R., Kupfer D.J. (1989). The Pittsburgh Sleep Quality Index: A new instrument for psychiatric practice and research. Psychiatry Res..

[35] Zung W.W. (1965). A Self-Rating Depression Scale. Arch. Gen. Psychiatry.

[36] Feng Q., Zhang Q.L., Du Y., Ye Y.L., He Q.Q. (2014). Associations of physical activity, screen time with depression, anxiety and sleep quality among Chinese college freshmen. PLoS ONE.

[37] Luo H., Xu X., Gao H., Zhang J., Zhang Z. (2021). Relationship of Anxiety and Depression with Perfectionism in Patients with Aesthetic All-Ceramic Repair of Anterior Teeth. Med. Sci. Monit..

[38] Shao R., He P., Ling B., Tan L., Xu L., Hou Y., Kong L., Yang Y. (2020). Prevalence of depression and anxiety and correlations between depression, anxiety, family functioning, social support and coping styles among Chinese medical students. BMC Psychol..

[39] Zung W.W., Richards C.B., Short M.J. (1965). Self-rating depression scale in an outpatient clinic. Further validation of the SDS. Arch. Gen. Psychiatry.

[40] Zhu Z., Cui Y., Gong Q., Huang C., Guo F., Li W., Zhang W., Chen Y., Cheng X., Wang Y. (2019). Frequency of breakfast consumption is inversely associated with the risk of depressive symptoms among Chinese university students: A cross-sectional study. PLoS ONE.

[41] Wild B., Eckl A., Herzog W., Niehoff D., Lechner S., Maatouk I., Schellberg D., Brenner H., Muller H., Lowe B. (2014). Assessing generalized anxiety disorder in elderly people using the GAD-7 and GAD-2 scales: Results of a validation study. Am. J. Geriatr. Psychiatry.

[42] Zhou S.J., Zhang L.G., Wang L.L., Guo Z.C., Wang J.Q., Chen J.C., Liu M., Chen X., Chen J.X. (2020). Prevalence and socio-demographic correlates of psychological health problems in Chinese adolescents during the outbreak of COVID-19. Eur. Child Adolesc. Psychiatry.

[43] Zhou S.J., Wang L.L., Yang R., Yang X.J., Zhang L.G., Guo Z.C., Chen J.C., Wang J.Q., Chen J.X. (2020). Sleep problems among Chinese adolescents and young adults during the coronavirus-2019 pandemic. Sleep Med..

[44] Sun J., Liang K., Chi X., Chen S. (2021). Psychometric Properties of the Generalized Anxiety Disorder Scale-7 Item (GAD-7) in a Large Sample of Chinese Adolescents. Healthcare.

[45] Spitzer R.L., Kroenke K., Williams J.B., Lowe B. (2006). A brief measure for assessing generalized anxiety disorder: The GAD-7. Arch. Intern. Med..

[46] Kroenke K., Spitzer R.L., Williams J.B., Monahan P.O., Lowe B. (2007). Anxiety disorders in primary care: Prevalence, impairment, comorbidity, and detection. Ann. Intern. Med..

[47] Zhou S.J., Wang L.L., Qi M., Yang X.J., Gao L., Zhang S.Y., Zhang L.G., Yang R., Chen J.X. (2021). Depression, Anxiety, and Suicidal Ideation in Chinese University Students During the COVID-19 Pandemic. Front. Psychol..

[48] Xu Y., Qi J., Yang Y., Wen X. (2016). The contribution of lifestyle factors to depressive symptoms: A cross-sectional study in Chinese college students. Psychiatry Res..

[49] Li W., Yin J., Cai X., Cheng X., Wang Y. (2020). Association between sleep duration and quality and depressive symptoms among university students: A cross-sectional study. PLoS ONE.

[50] Li Y., Li G.X., Yu M.L., Liu C.L., Qu Y.T., Wu H. (2021). Association Between Anxiety Symptoms and Problematic Smartphone Use Among Chinese University Students: The Mediating/Moderating Role of Self-Efficacy. Front. Psychiatry.

[51] Zou P., Sun L., Yang W., Zeng Y., Chen Q., Yang H., Zhou N., Zhang G., Liu J., Li Y. (2018). Associations between negative life events and anxiety, depressive, and stress symptoms: A cross-sectional study among Chinese male senior college students. Psychiatry Res..

[52] Yang J., Peek-Asa C., Corlette J.D., Cheng G., Foster D.T., Albright J. (2007). Prevalence of and risk factors associated with symptoms of depression in competitive collegiate student athletes. Clin. J. Sport Med..

[53] Li H., Moreland J.J., Peek-Asa C., Yang J. (2017). Preseason Anxiety and Depressive Symptoms and Prospective Injury Risk in Collegiate Athletes. Am. J. Sports Med..

[54] Gehrman P., Seelig A.D., Jacobson I.G., Boyko E.J., Hooper T.I., Gackstetter G.D., Ulmer C.S., Smith T.C. (2013). Predeployment Sleep Duration and Insomnia Symptoms as Risk Factors for New-Onset Mental Health Disorders Following Military Deployment. Sleep.

[55] Szklo-Coxe M., Young T., Peppard P.E., Finn L.A., Benca R.M. (2010). Prospective associations of insomnia markers and symptoms with depression. Am. J. Epidemiol..

[56] Zhai L., Zhang H., Zhang D. (2015). Sleep Duration and Depression among Adults: A Meta-Analysis of Prospective Studies. Depress. Anxiety.

[57] Lin W., Bai G., He W., Yang F., Li W., Min Y., Lu Y., Hsing A., Zhu S. (2021). Association between napping status and depressive symptoms in urban residents during the COVID-19 epidemic. Zhejiang Da Xue Xue Bao Yi Xue Ban.

[58] Kim H.M., Lee S.W. (2018). Beneficial Effects of Appropriate Sleep Duration on Depressive Symptoms and Perceived Stress Severity in a Healthy Population in Korea. Korean J. Fam. Med..

[59] Racic M., Todorovic R., Ivkovic N., Masic S., Joksimovic B., Kulic M. (2017). Self-Perceived Stress in Relation to Anxiety, Depression and Health-related Quality of Life among Health Professions Students: A Cross-sectional Study from Bosnia and Herzegovina. Zdr. Varst..

[60] Carskadon M.A., Sharkey K.M., Knopik V.S., McGeary J.E. (2012). Short sleep as an environmental exposure: A preliminary study associating 5-HTTLPR genotype to self-reported sleep duration and depressed mood in first-year university students. Sleep.

[61] Fava M. (2004). Daytime sleepiness and insomnia as correlates of depression. J. Clin. Psychiatry.

[62] Vgontzas A.N., Papanicolaou D.A., Bixler E.O., Kales A., Tyson K., Chrousos G.P. (1997). Elevation of plasma cytokines in disorders of excessive daytime sleepiness: Role of sleep disturbance and obesity. J. Clin. Endocrinol. Metab..

